# Supplementary material for: Pyrolytic Behavior of Major Biomass Components in Waste Biomass
Source: Polymers (Basel). 2019 Feb 13;11(2):324. doi: 10.3390/polym11020324 (PMC6419179; doi:10.3390/polym11020324)
Supplement: Supplementary file 1 [file polymers-11-00324-s001.pdf]

Supporting information

Pyrolytic behavior of major biomass components in the waste biomasses

Haoxi Ben [1,2,*], Zhihong Wu [1,2], Guangting Han [3,4], Wei Jiang [3,4] and Arthur Ragauskas [5,6]

[1] Southeast University, Nanjing, China;

[2] Key Laboratory of Energy Thermal Conversion and Control of Ministry of Education, Nanjing, China;

[3] Qingdao University, Qingdao, China;

[4] State Key Laboratory of Bio-Fibers and Eco-Textiles, Qingdao University, Qingdao, China

[5] Joint Institute for Biological Sciences, Biosciences Division, Oak Ridge National Lab, Oak Ridge, TN 37831, USA;

[6] Department of Chemical and Biomolecular Engineering, University of Tennessee, Knoxville, Tennessee 37996, USA

[*]Correspondence should be addressed to Dr. Haoxi Ben

Email: benhaoxi@gmail.com

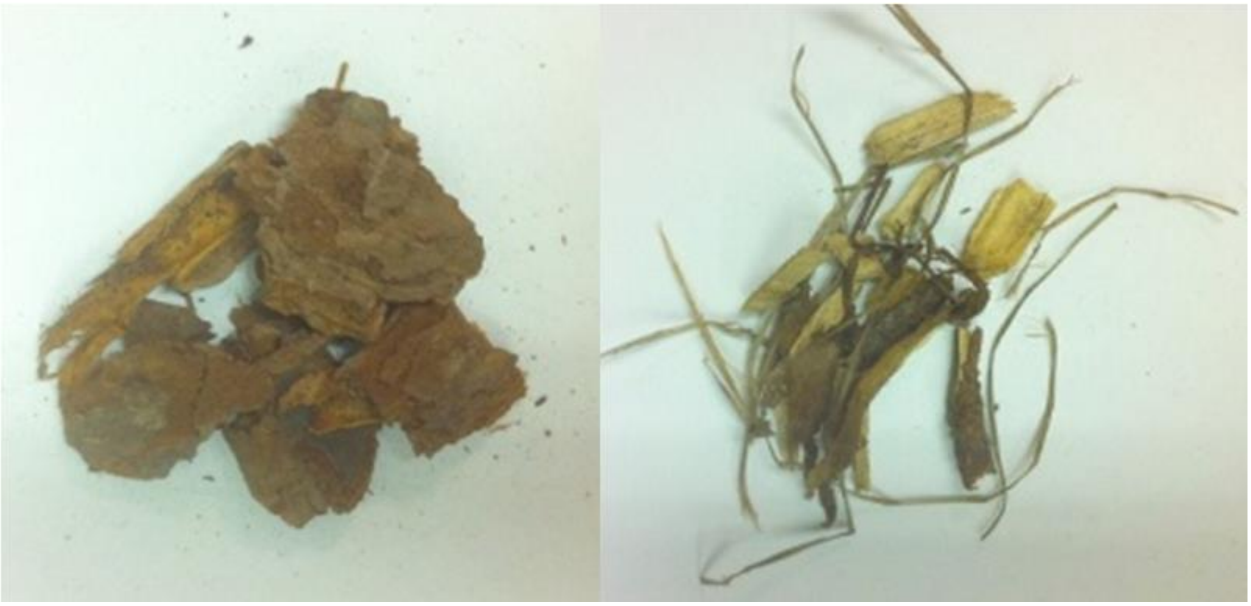

Figure S1. Pine bark (left) and pine residues (right) used in this study.

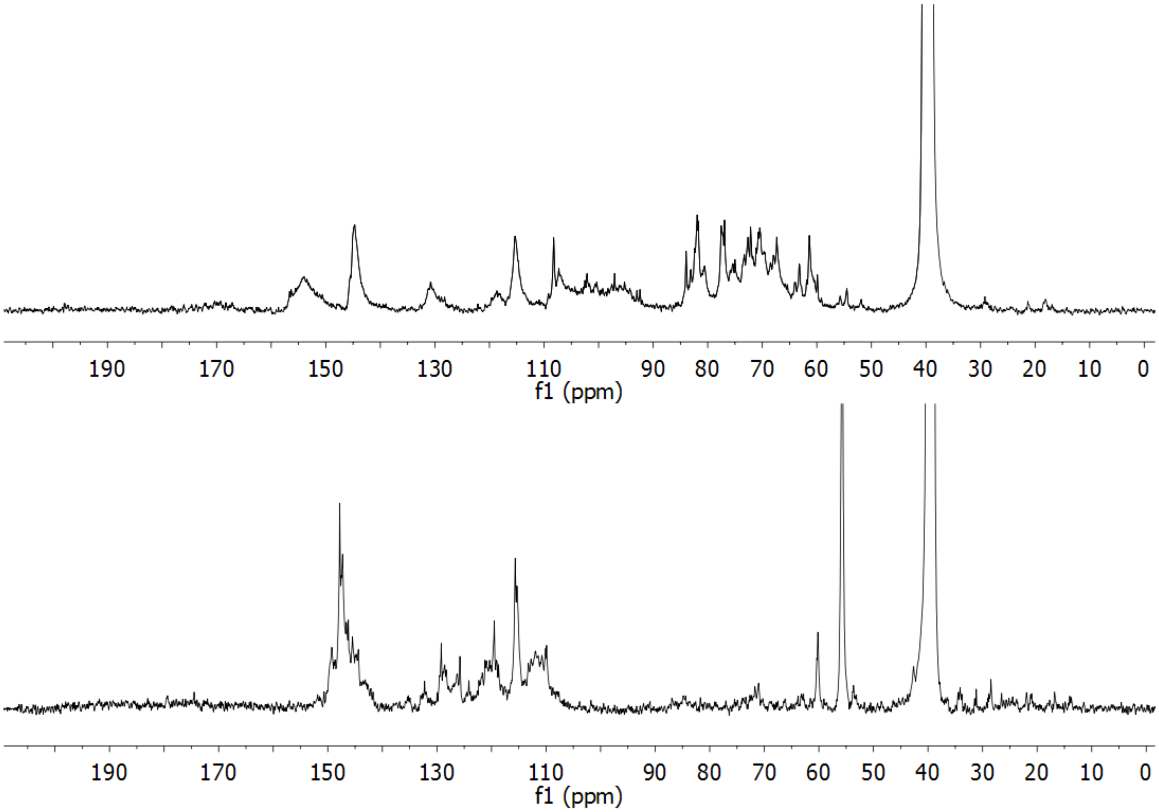

Figure S2. Quantitative ^{13}C NMR for the tannin (top) and lignin (bottom) used in this study.

Table S1. Quantitative ^{13}C NMR results for the tannin and lignin used in this study.

Biomass components	-**C**=O bonds	Aromatic **C**-O bonds	Aromatic **C**-C bonds	Aromatic **C**-H bonds	Aliphatic **C**-O bonds	Methoxyl groups	Aliphatic **C**-C bonds
Tannin	1.9%	15.6%	3.6%	28.0%	50.3%	-	0.7%
Lignin	6.4%	25.7%	9.6%	31.3%	8.5%	14.0%	4.5%

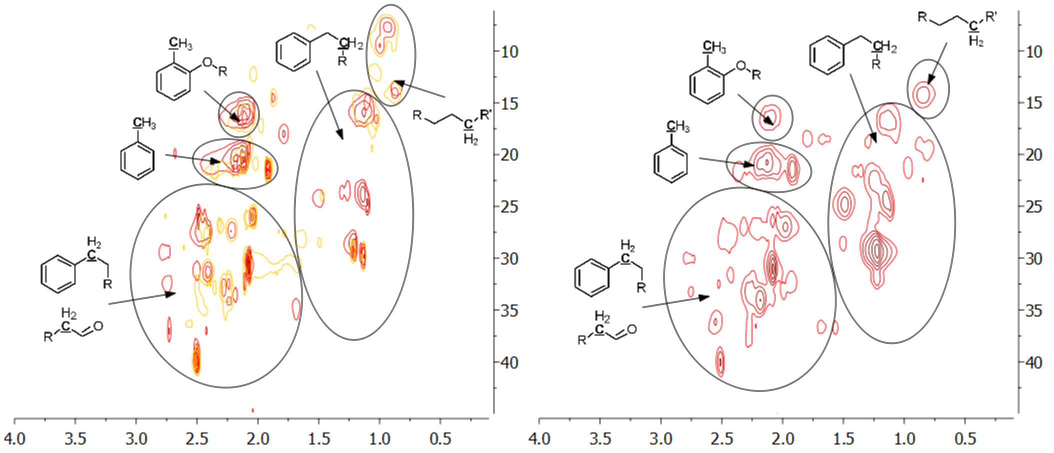

Figure S3a. Aliphatic C-H bonds in the HSQC-NMR spectra for artificial mixture of pyrolysis oils produced from lignin, tannin, cellulose and hemicellulose (left) and pine bark pyrolysis oil (right) produced at 600 °C.

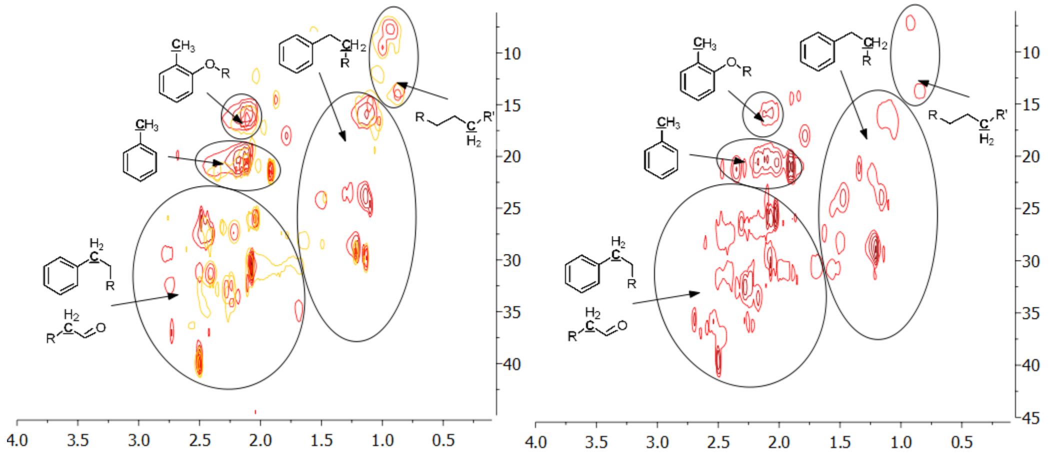

Figure S3b. Aliphatic C-H bonds in the HSQC-NMR spectra for artificial mixture of pyrolysis oils produced from lignin, tannin, cellulose and hemicellulose (left) and pine residue pyrolysis oil (right) produced at 600 °C.

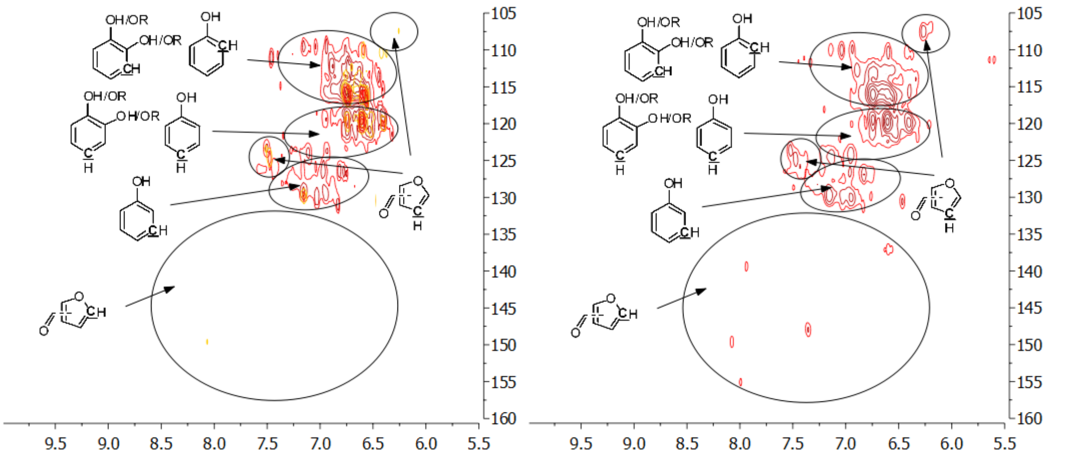

Figure S4a. Aromatic C-H bonds in the HSQC-NMR spectra for artificial mixture of pyrolysis oils produced from lignin, tannin, cellulose and hemicellulose (left) and pine bark pyrolysis oil (right) produced at 600 °C.

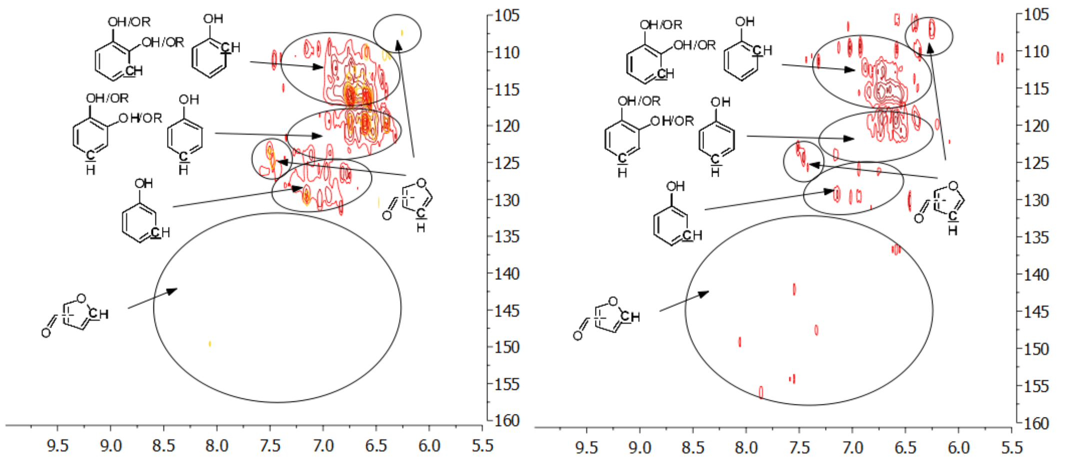

Figure S4b. Aromatic C-H bonds in the HSQC-NMR spectra for artificial mixture of pyrolysis oils produced from lignin, tannin, cellulose and hemicellulose (left) and pine residue pyrolysis oil (right) produced at 600 °C.

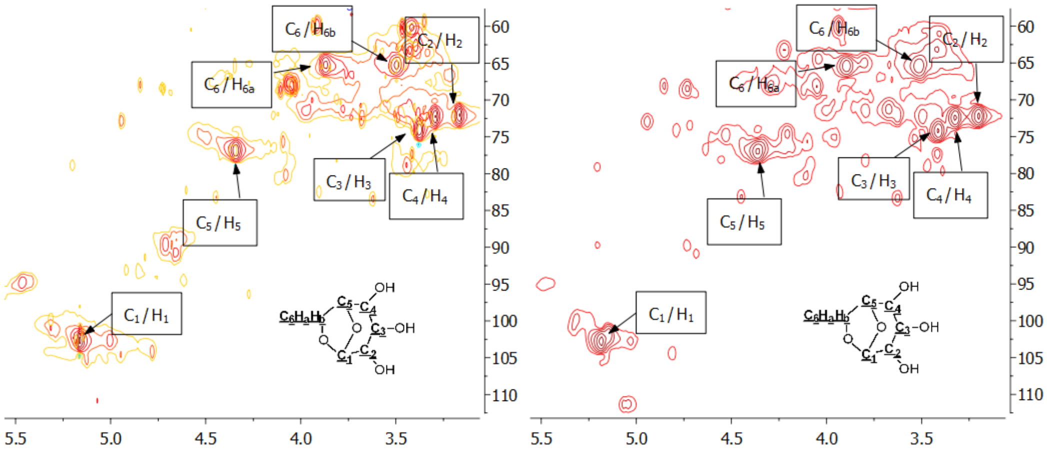

Figure S5a. Levoglucosan in the HSQC-NMR spectra for artificial mixture of pyrolysis oils produced from lignin, tannin, cellulose and hemicellulose (left) and pine bark pyrolysis oil (right) produced at 600 °C.

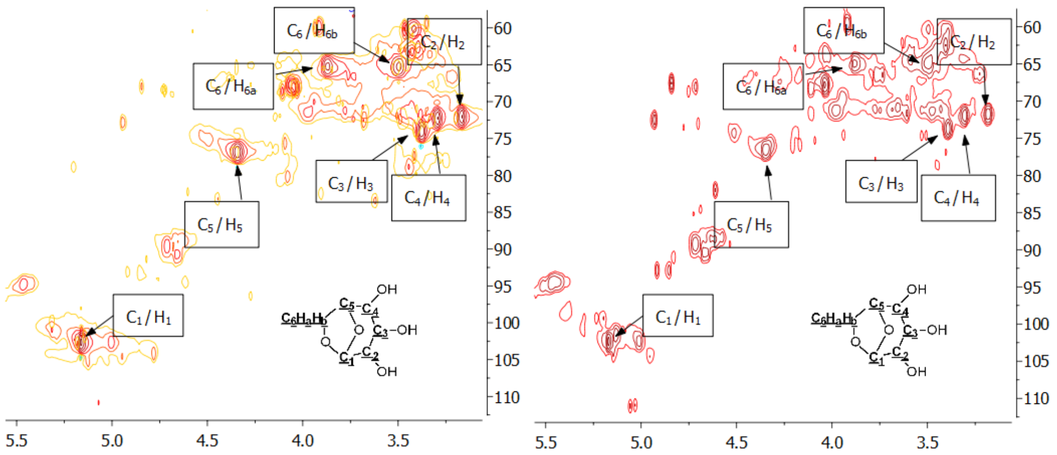

Figure S5b. Levoglucosan in the HSQC-NMR spectra for artificial mixture of pyrolysis oils produced from lignin, tannin, cellulose and hemicellulose (left) and pine residue pyrolysis oil (right) produced at 600 °C.

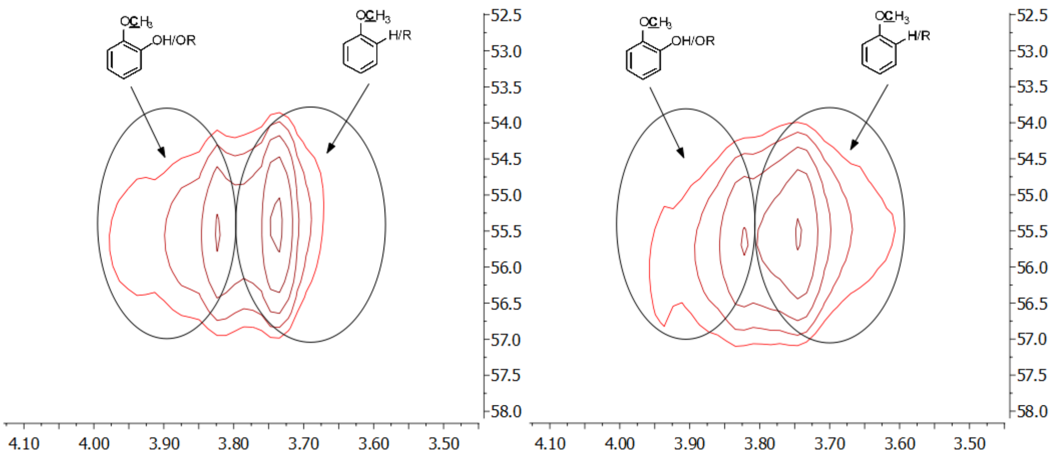

Figure S6a. Methoxyl groups in the HSQC-NMR spectra for artificial mixture of pyrolysis oils produced from lignin, tannin, cellulose and hemicellulose (left) and pine bark pyrolysis oil (right) produced at 600 °C.

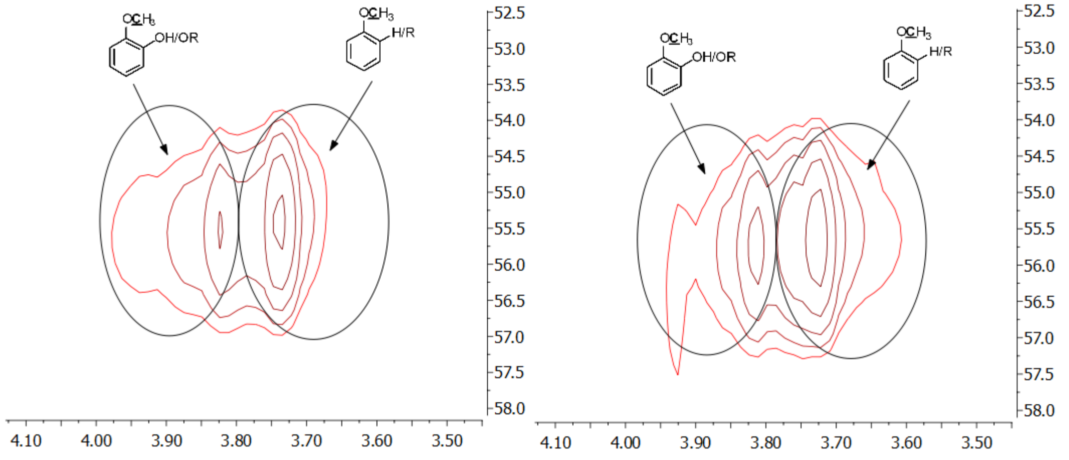

Figure S6b. Methoxyl groups in the HSQC-NMR spectra for artificial mixture of pyrolysis oils produced from lignin, tannin, cellulose and hemicellulose (left) and pine residue pyrolysis oil (right) produced at 600 °C.

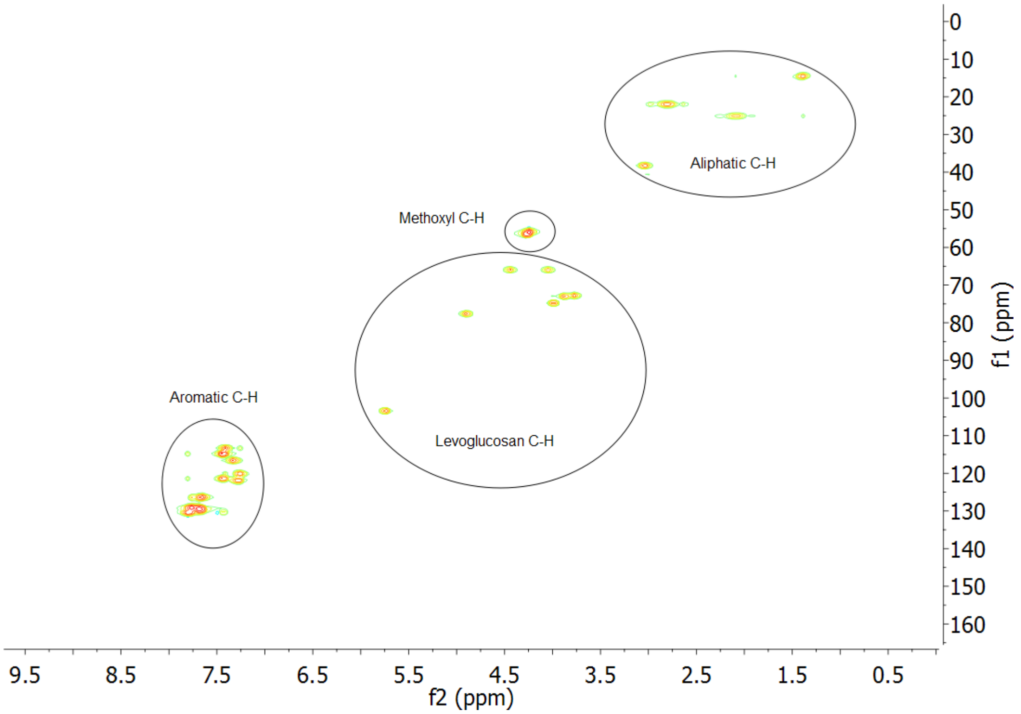

Figure S7. HSQC-NMR for standard mixtures of pyrolysis oil model compounds.

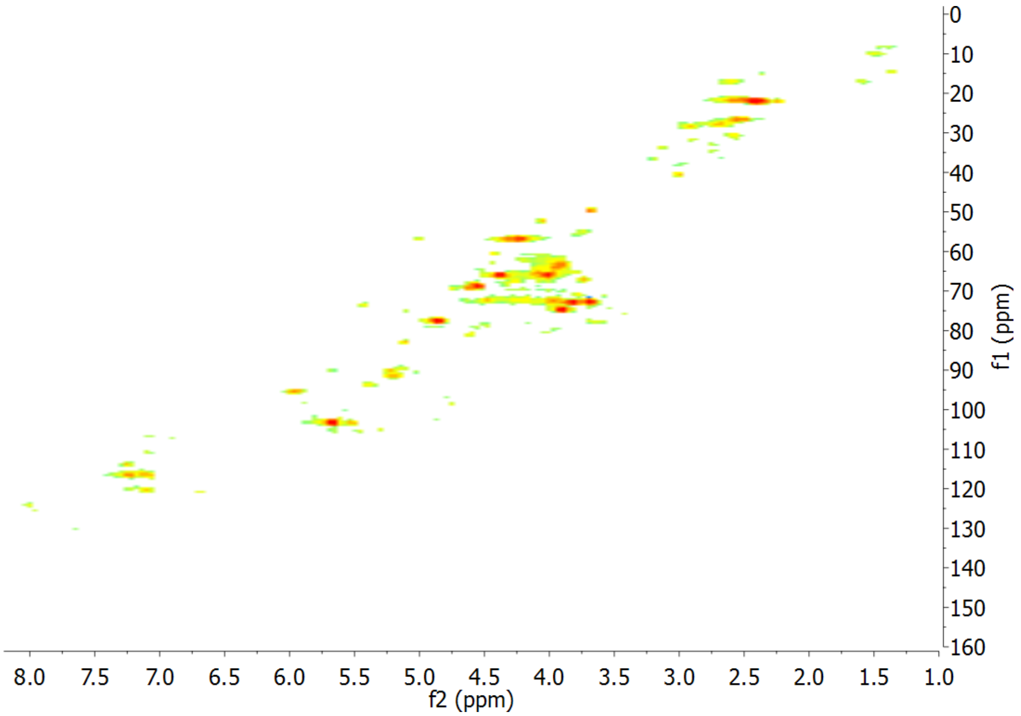

Figure S8. HSQC-NMR for pyrolysis oil produced from a pilot plant reactor.

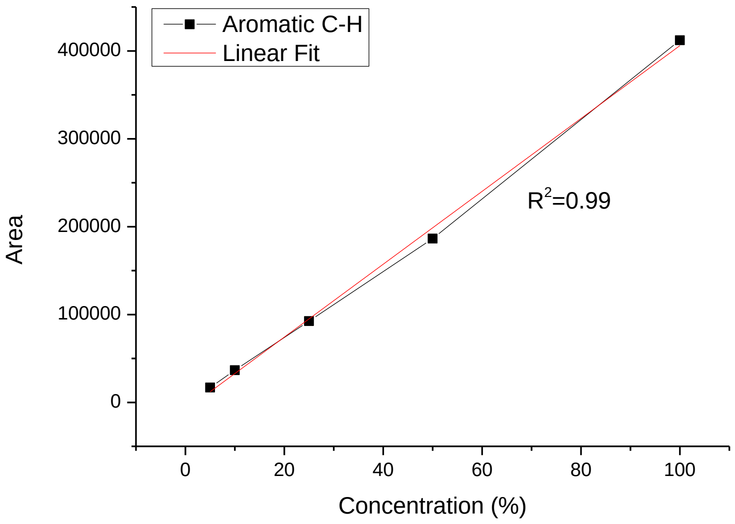

Figure S9a. Integrations and linear fit for aromatic C-H bonds in the HSQC-NMR for standard mixtures (100%, 50%, 25%, 10%, and 5% concentrations) of pyrolysis oil model compounds.

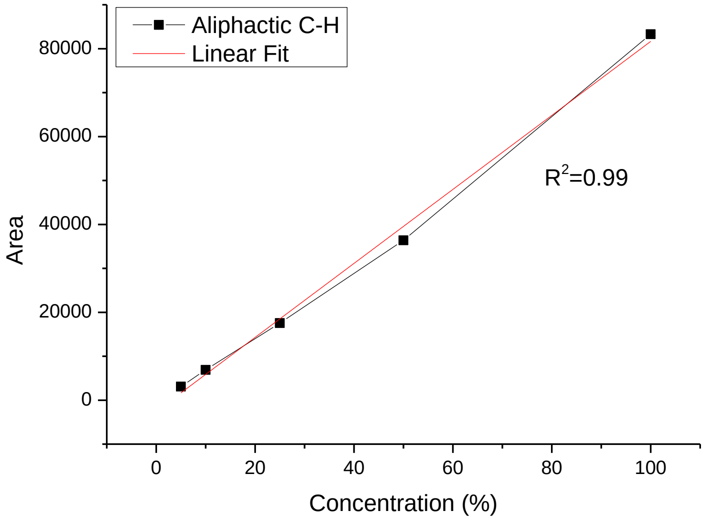

Figure S9b. Integrations and linear fit for aliphatic C-H bonds in the HSQC-NMR for standard mixtures (100%, 50%, 25%, 10%, and 5% concentrations) of pyrolysis oil model compounds.

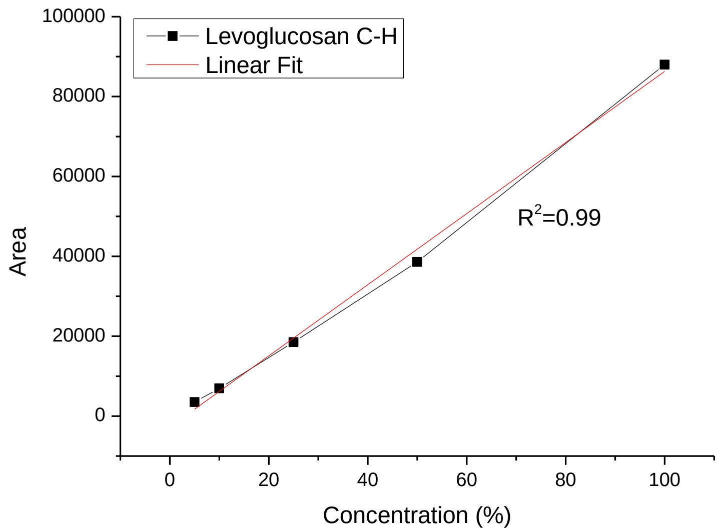

Figure S9c. Integrations and linear fit for levoglucosan C-H bonds in the HSQC-NMR for standard mixtures (100%, 50%, 25%, 10%, and 5% concentrations) of pyrolysis oil model compounds.

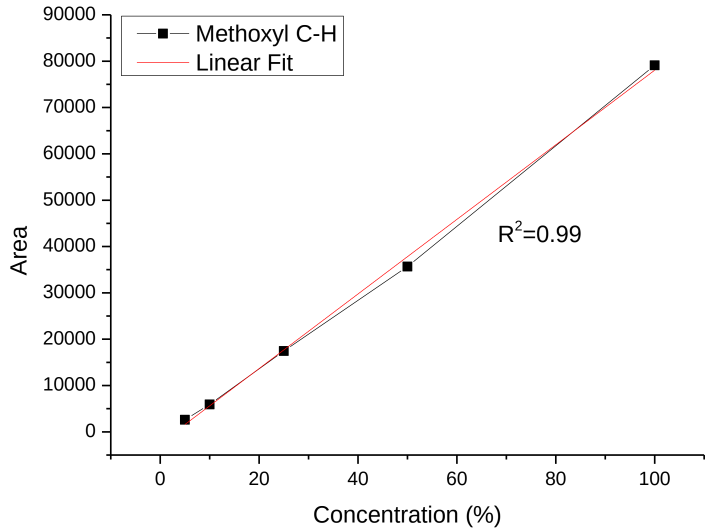

Figure S9d. Integrations and linear fit for aliphatic C-H bonds in the HSQC-NMR for standard mixtures (100%, 50%, 25%, 10%, and 5% concentrations) of pyrolysis oil model compounds.

Table S2. Functional groups recover ratio for artificial mixtures of pyrolysis oil (produced from a pilot plant reactor) and pyrolysis oil model compounds based on HSQC-NMR.

Functional groups	Pyrolysis oil + guaiacol	Pyrolysis oil + levoglucosan	Pyrolysis oil + propylbenzene
Aromatic C-H	95% *	-	98%
Methoxy C-H	99%	-	-
Levoglucosan C-H	-	100%	-
Aliphatic C-H	-	-	103%

* Recover ratio= (mol of functional groups in the artificial mixtures) / (calculated mol of functional groups based on calibration curves for difference functional groups in HSQC-NMR)

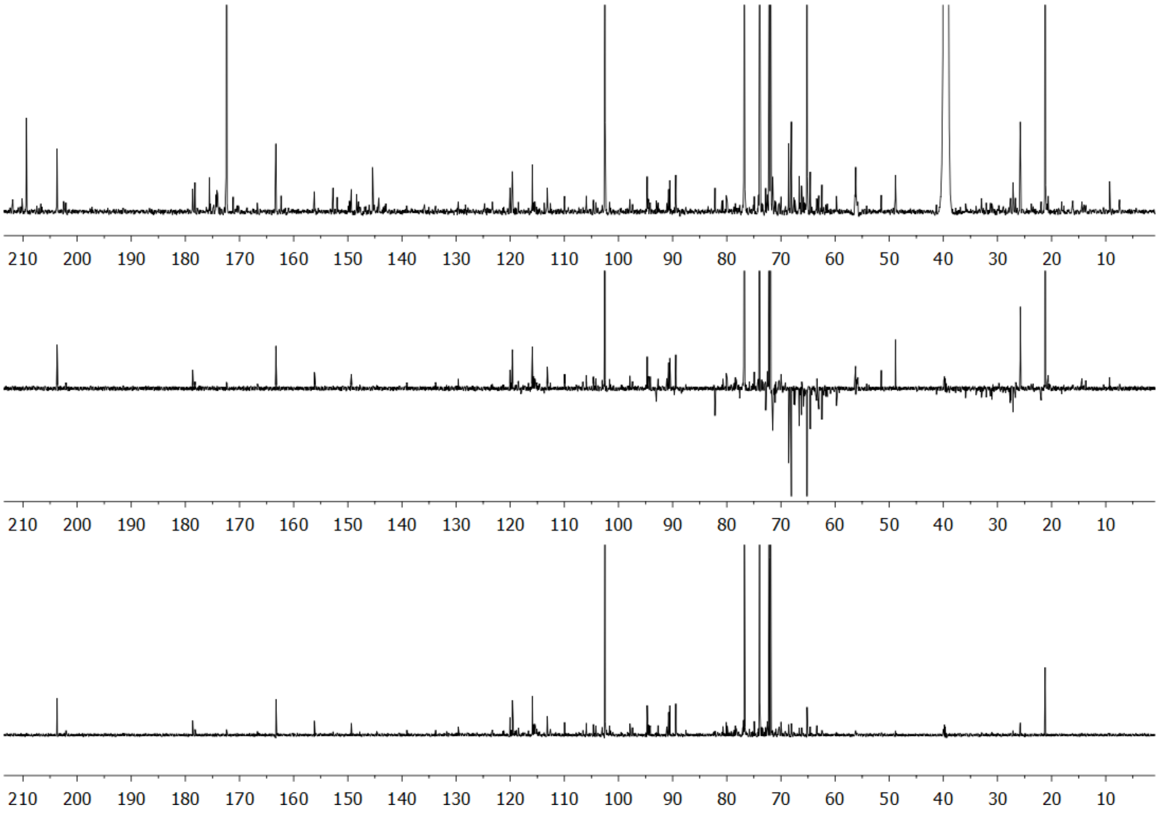

Figure S10. Quantitative ^{13}C NMR and DEPT 135 and DEPT 90 (from top to bottom) for the pyrolysis oil produced from a pilot plant reactor.

Table S3. ^{13}C-NMR chemical shift assignment ranges for pyrolysis oil (on the basis of reference 1)

Type of carbons	Range (ppm)	Structure
Carbonyl	215.0 – 166.5	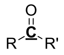
Aromatic C-O	166.5 – 142.0	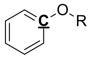
Aromatic C-C	142.0 – 125.0	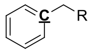
Aromatic C-H	125.0 – 95.8	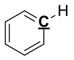
Levoglucosan	C1 102.3, C2 72.0<br>C3 73.7, C4 71.7<br>C5 76.5, C6 64.9	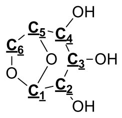
Aliphatic C-O	95.8 – 60.8	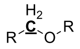
Methoxyl	60.8 – 55.2	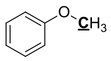
Aliphatic C-C	55.2 – 0.0	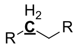
Methyl – Aromatic	21.6 – 19.1	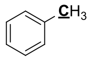
Methyl – Aromatic’	16.1 – 15.4	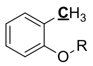

<u>Commercial Standard mixtures</u>

The evaluation for the quantitative ^{13}C NMR has been done for two commercial standard samples. The contents for different components in these two commercial standards have been summarized in Table S4 and S5.

Table S4. Certified value for standard #1.

Compound	Certified Value (wt %)	Formula
cyclopentane	1.001	c-C5H10
n-pentane	0.99	n-C5H12
cyclohexane	2.003	c-C5H12
2,3-dimethylbutane	1.98	C6H14

n-hexane	2.03	C6H14
1-hexene	1.503	C6H12
4-methyl-1-hexene	1.493	C7H14
benzene	2.248	C6H6
methylcyclohexane	4.243	C7H14
n-heptane	3.479	C7H16
toluene	2.254	C7H8
2,2,4-trimethylpentane	4.99	C8H18
n-octane	4.999	C8H18
1,2-dimethylcyclohexane	4.948	C8H16
trimethylcyclohexane isomer	0.595	C9H18
ethylbenzene	4.46	C8H10
1,2,4-trimethylcyclohexane	3.652	C9H18
o-xylene	4.207	C8H10
n-nonane	4.488	C9H20
n-propylbenzene	4.998	C9H12
1,2,4-trimethylbenzene	4.401	C9H12
n-decane	4.229	C10H22
1,2,3-trimethylbenzene	4.217	C9H12
trans-decahydronaphthalene	4.211	C8H18
n-undecane	3.464	C11H24
1,2,4,5-tetramethylbenzene	4.812	C10H14
n-dodecane	3.246	C12H26
pentamethylbenzene	4.999	C11H16
n-tetradecane	4.423	C14H30
sum	98.5626	

Table S5. Certified value for standard #2.

Compound	Certified Value (wt %)	Formula
n-pentane	6.55	C5H12
n-hexane	6.62	C6H14
n-heptane	6.64	C7H16
n-octane	6.49	C8H18
n-nonane	6.61	C9H20
n-decane	6.66	C10H22
n-undecane	6.59	C11H24
n-dodecane	13.3	C12H26
n-tetradecane	6.66	C14H30
n-pentadecane	6.63	C15H32
n-hexadecane	6.62	C16H34
n-heptadecane	6.61	C17H36
n-octadecane	6.66	C18H38
n-eicosane	6.65	C20H42
sum	99.29	

Two evaluations have been examined for two standard mixtures. Both of these two evaluations (Tables S6 and S7) for the ^{13}C NMR of complex mixtures approved that the ^{13}C NMR method used in this study can provide quantitative results for complicated mixtures.

Table S6. Evaluations for quantitative ^{13}C NMR of standard #1.

Calculation methods	Functional groups (carbon mol%)		
	Aliphatic carbons	Aromatic carbons	Double bonds
Based on the concentrations listed in Table 2	72.42	27.08	0.51
Based on NMR data	70.74	28.70	0.55

Calculation methods	Different types of carbons (carbon mol%)			
	C	CH	CH2	CH3
Based on the concentrations listed in Table 2	10.77	22.88	40.87	25.48
Based on NMR data	10.22	21.58	41.34	26.84

Table S7. Evaluations for quantitative ^{13}C NMR of standard #2.

Calculation methods	Different types of carbons (carbon mol%)			
	C	CH	CH2	CH3
Based on the concentrations listed in Table 3	0	0	19.49	80.51
Based on NMR data	0	0	19.05	80.95

Table S8. T1 value for different functional groups in pyrolysis oil produced from a pilot plant reactor when employing 5mg/ml $Cr(acac)_3$ as relax reagent.

	**C**=O	Aromatic carbons	Aliphatic **C**-O	Aliphatic **C**-C
	230 - 166.5 ppm	166.5 - 95.8 ppm	95.8 - 53.5 ppm	35.3 - 0.0 ppm
5mg/ml $Cr(acac)_3$	459.268ms	211.380ms	277.742ms	269.905ms

Table S9. Integrations for each 1mmol carbon in the functional groups calculated by analysis for artificial mixtures of pyrolysis oil (produced from a pilot plant reactor) and pyrolysis oil model compounds based on quantitative ^{13}C NMR.

Integrations	Pyrolysis oil + guaiacol	Pyrolysis oil + levoglucosan	Pyrolysis oil + propylbenzene
Area/1mmol carbon in the functional groups	4600346 *	4235643	4977048

* Area/ 1mmol carbon in the functional groups=((integration area for (pyrolysis oil+ guaiacol (or other model compounds)) – (integration area for pyrolysis oil (the same amount in the artificial mixtures)))/(mmol of guaiacol (or other model compounds used in the artificial mixtures)*(number of carbons in the guaiacol molecule))

Table S10. Comparisons of quantitative ^{13}C NMR and HSQC-NMR investigations on pyrolysis oil sample (a pilot plant product).

Functional groups	Calculated by quantitative ^{13}C NMR	Functional groups	Calculated by HSQC NMR
	µmol (carbons)/mg pyrolysis oil		µmol (C-H bonds)/mg pyrolysis oil
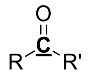	1.21		
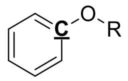	0.95		
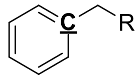	0.03		
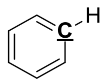	2.07	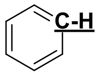	2.06 *

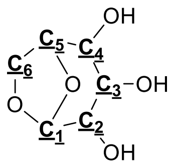	6.04	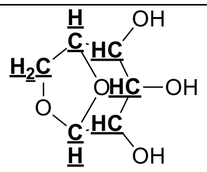	5.77 *
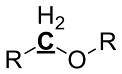	7.84	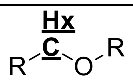	11.59 #
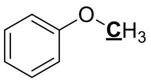	0.50	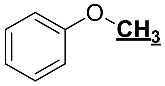	0.50 *
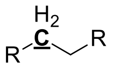	2.58	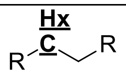 X=~2.5-2.6 &	6.46 #
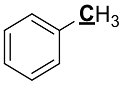	0.02		
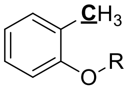	0.08		

* The contents for these C-H bonds have been converted to the contents based on the carbon numbers in these functional groups.

# The contents for these C-H bonds have not been converted to the contents based on the carbon numbers in these functional groups.

& X=6.46 (μmol/mg for each C-H)/2.58(μmol/mg for the carbon in this functional group) =2.5

The X has also been proposed based on the quantitative ^{13}C NMR and DEPT 135 (CH3 and CH have positive peaks and CH2 have negative peaks) and DEPT 90 NMR (only shows the CH as positive peaks). X= (integration area for aliphatic CH3 peaks*3 + integration area for aliphatic CH2 peaks*2 + integration area for aliphatic CH peaks *1)/(integration area for all the aliphatic peaks). The X=2.6 based on the quantitative ^{13}C NMR and DEPT 135 and DEPT 90 NMR.

Table S11. ^{1}H and ^{13}C NMR chemical shift assignment for the compounds reported present in tannin pyrolysis oils.

Compound	Structure and chemical shift assignments
phenol	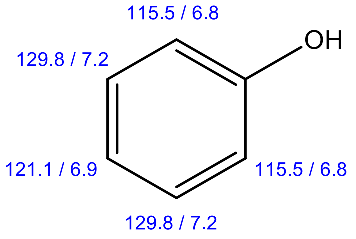
4-methylphenol<br><br>p-cresol	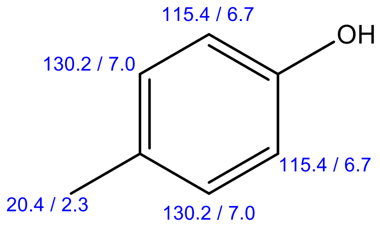
m-cresol<br><br>phenol, 3-methyl-	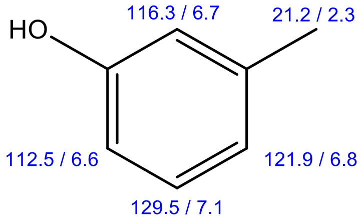
2-methoxyphenol<br><br>phenol, 2-methoxy-<br><br>guaiacol	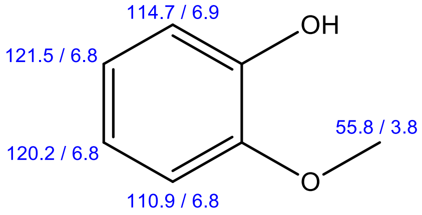
2,6-dimethylphenol<br><br>phenol, 2,6-dimethyl-<br><br>2,6-xylenol	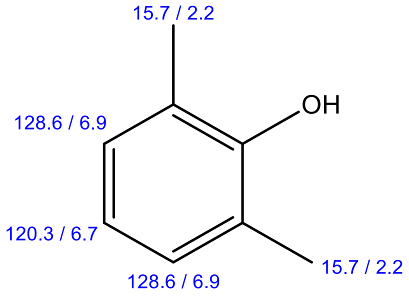

2,3-dimethylphenol

phenol, 2,3-dimethyl-

2,3-xylenol

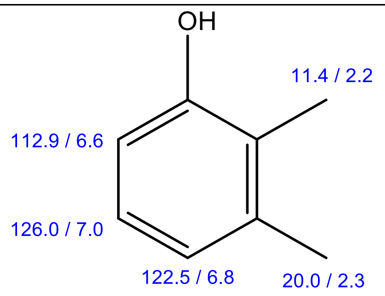

---

benzoic acid

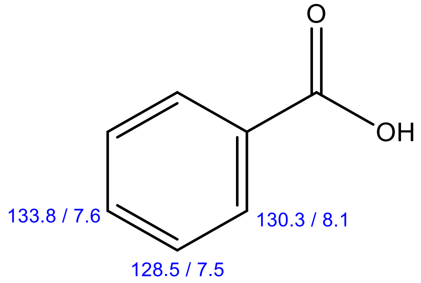

2-methoxy-4-methylphenol

phenol, 2-methoxy-4-methyl-

4-methylguaiacol

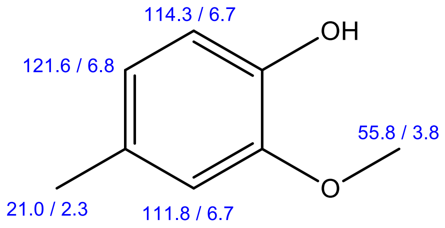

---

catechol

1,2-benzenediol

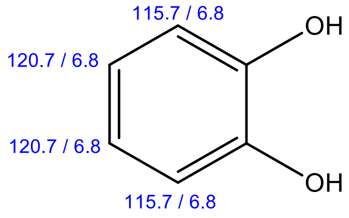

4-Methoxyphenol

mequinol

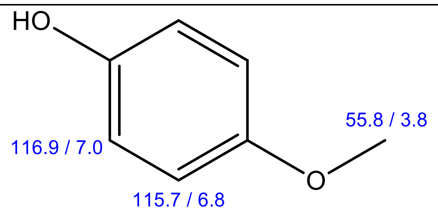

4-methyl-1,2-benzenediol

1,2-benzenediol, 4-methyl-

4-methylcatechol

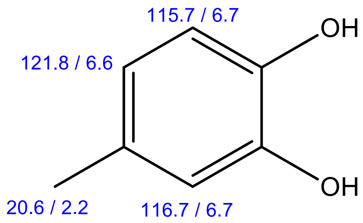

Hydroquinone<br>benzene-1,4-diol	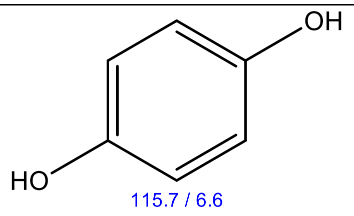
2-methoxy-4-ethylphenol<br>phenol, 4-ethyl-2-methoxy-	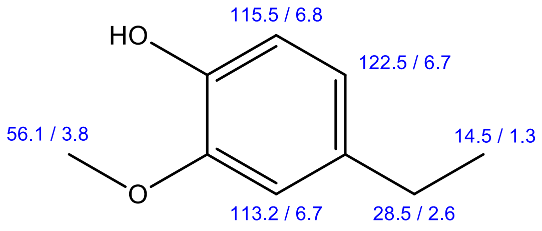
Benzene-1,3-diol<br>resorcinol	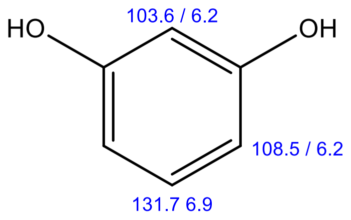
1,3-benzenediol, 2-methyl-	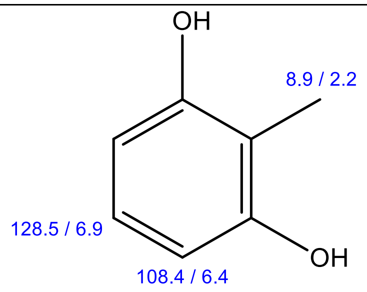
1,4-benzenediol, 2-methyl-	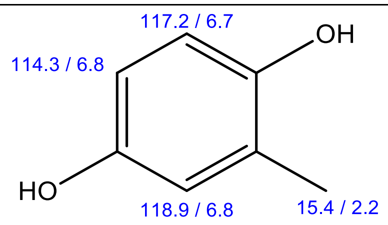
1,2,3-benzenetriol<br>pyrogallol	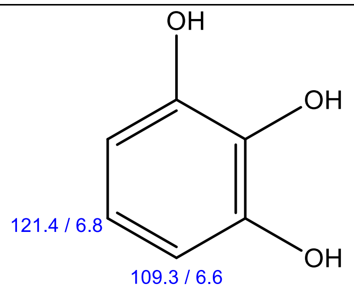

4-ethyl-1,3-benzenediol<br>1,3-benzenediol, 4-ethyl-<br>4-ethylresorcinol	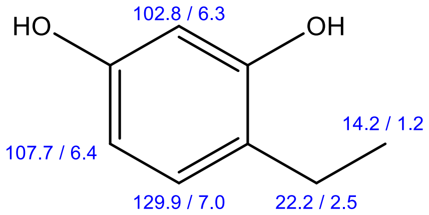
1,4-benzenediol, 2-methoxy-	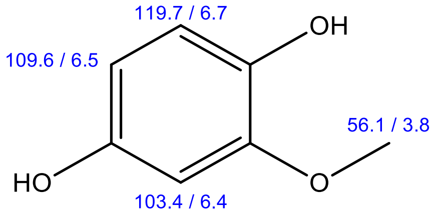
benzoic acid, 3,4-dihydroxy-, methyl ester	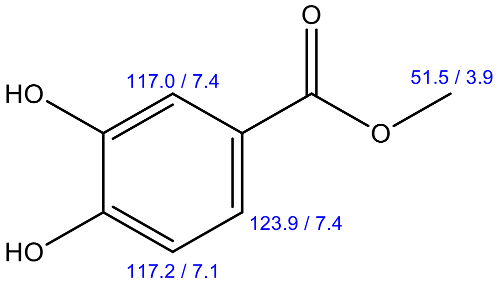

Table S12. ^{13}C NMR chemical shift assignment for the compounds reported present in hemicellulose pyrolysis oils. The compounds in this database are based on three references. [2-4]

Compound	Structure and chemical shift assignments
acetaldehyde	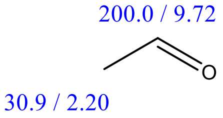
formic acid	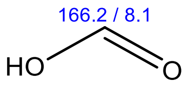
acetic acid	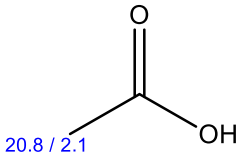

2-methylfuran	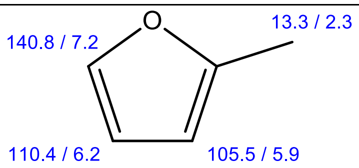
2-hydroxyacetic acid	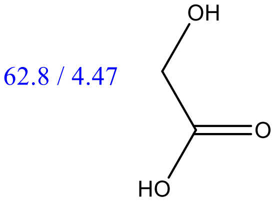
(2R,3R,4S,5R)-tetrahydro-2H-pyran-2,3,4,5-tetraol	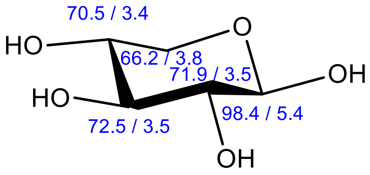
furfural<br><br>2-furaldehyde	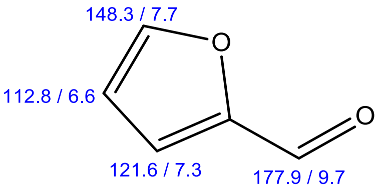
2,7-dioxabicyclo[2.2.1]heptane-5,6-diol	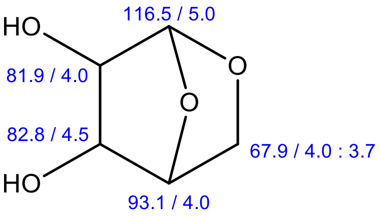
3,4-dihydroxytetrahydrofuran-2-carbaldehyde	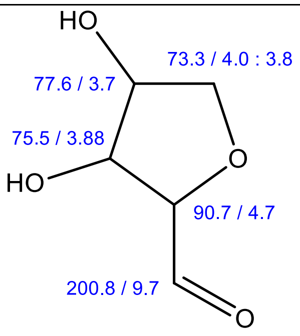

5-hydroxy-2H-pyran-4(3H)-one	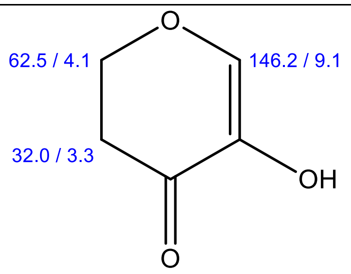
4-hydroxy-5,6-dihydro-2H-pyran-2-one	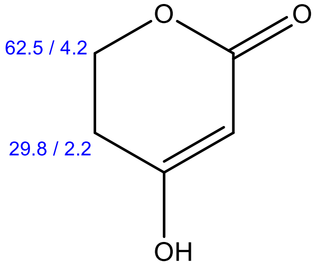
2,7-dioxabicyclo[2.2.1]heptan-5-one	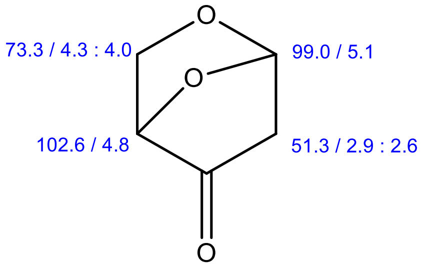
2,7-dioxabicyclo[2.2.1]heptan-6-one	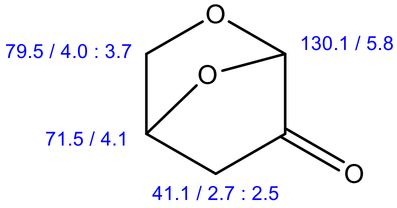
1-hydroxy-2-propanone<br><br>hydroxyacetone	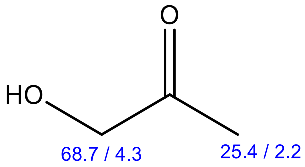
1-Hydroxy-2-butanone	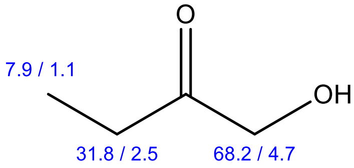
Pentanal	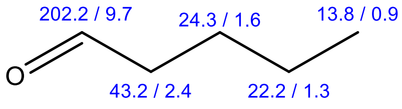

1,2-Cyclopentanedione	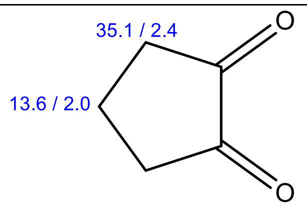
2-Hydroxy-1-methylcyclopenten-3-one	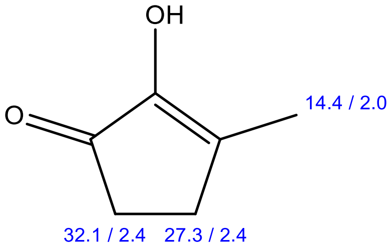
3-Ethyl-2-hydroxy-2-cyclopenten-1-one	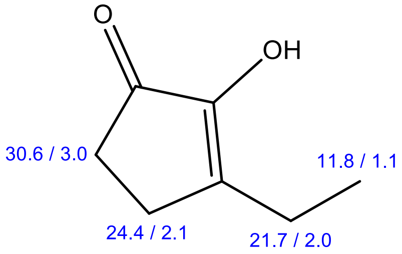
2-Ethyl-5-methyl-phenol	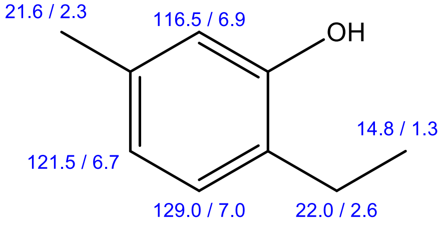
2,5-Dimethyl-phenol	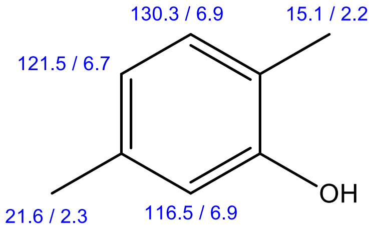
Acetoxyacetone	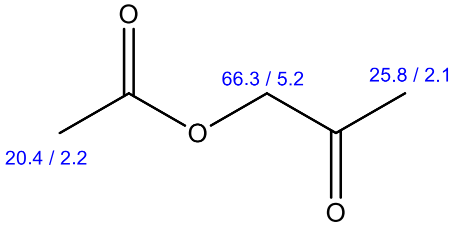

methanol	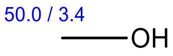
acetone	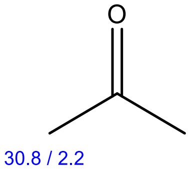

References

1. Ben, H.; Ragauskas, A. J., *Energy Fuels* **2011,** 25, (5), 2322-2332.

2. Patwardhan, P. R.; Brown, R. C.; Shanks, B. H., *ChemSusChem* **2011,** 4, (5), 636-643.

3. Peng, Y.; Wu, S., *J. Anal. Appl. Pyrolysis* **2010,** 88, (2), 134-139.

4. Shen, D. K.; Gu, S.; Bridgwater, A. V., *J. Anal. Appl. Pyrolysis* **2010,** 87, (2), 199-206.
